# Differences between natural sleep and the anesthetic state

**DOI:** 10.2144/fsoa-2020-0149

**Published:** 2020-11-30

**Authors:** Akshay Date, Khayam Bashir, Aaliya Uddin, Chandni Nigam

**Affiliations:** 1 Basildon & Thurrock University Hospital, Nethermayne, Basildon, Essex SS16 5NL, UK; 2 Kings College Hospital, Denmark Hill, Brixton, London SE5 9RS, UK

**Keywords:** anesthesia, differences, sleep

## Abstract

The anesthetic state and natural sleep share many neurobiological features and yet are two distinct states. The hallmarks of general anesthesia include hypnosis, analgesia, akinesia and anxiolysis. These are the principal parameters by which the anesthetic state differs from natural sleep. These properties are mediated by systemic administration of a combination of agents producing balanced anesthesia. The exact nature of anesthetic narcosis is dose dependent and agent specific. It exhibits a relative lack of nociceptive response and active suppression of motor and autonomic reflexes. Surgical anesthesia displays a signature electroencephalogram pattern of burst suppression that differs from rapid eye movement sleep, representing more widespread disruption of thalamocortical connectivity, impairing information integration and processing. These differences underpin successful anesthetic action. This review explores the differences between natural sleep and anesthetic-induced unconsciousness as induced by balanced anesthesia.

## Lay abstract

The anesthetic state and natural sleep share many similar features yet are two distinct states. The key features of general anesthesia are mediated by systemic administration of a combination of agents. The exact nature of anesthesia is dose dependent and agent specific. Anesthesia displays a more widespread disruption of brain connectivity compared with natural sleep, impairing information integration and processing. These differences are essential to successful anesthetic action. This review explores the differences between natural sleep and anesthetic-induced unconsciousness.

The anesthetic state refers to a pharmacologically induced reversible state of unconsciousness associated with physiological endpoints of analgesia, amnesia and akinesia [1]. There is also concomitant depression of ventilation and the autonomic nervous system. Few anesthetic agents individually fulfill all these physiological traits; hence, modern practice largely centers around the administration of combinations of agents that together achieve the desired anesthetic effect, while aiming to minimize side effects [2]. Sleep is endogenously generated involving the active suppression of consciousness by nuclei in the brainstem, diencephalon and basal forebrain [3]. There is periodic cycling between two distinct states in electroencephalogram (EEG) activity: rapid eye movement (REM) and non-rapid eye movement (NREM) sleep [4]. A large body of evidence suggests mechanistic similarities between anesthetic-induced unconsciousness and sleep.

Neuroanatomically, there is parallel between the GABA-mediated inhibition of arousal-promoting brain nuclei during sleep and general anesthesia [5]. In addition, EEG studies show similar brain activity during the anesthetized state and NREM sleep, although with some notable differences as exhibited by MacIver and Bland in the EEG results of rats anesthetized with isoflurane compared with slow-wave sleep. Both groups displayed similar high-amplitude delta activity alongside a 1- to 3-Hz dominant frequency; however isoflurane-induced delta activity lacked the higher frequencies seen during sleep [6]. Anesthetics that act through GABA_A_ receptors, for example propofol, are associated with beta oscillations; conversely, beta oscillations are seen to decrease in NREM sleep [7]. Another point to note in the case of propofol, general anesthesia has been shown to reverse sleep debt [8]. It should be said, however, that this phenomenon is not replicated across all types of anesthesia and is in fact contradicted with reference to volatile anesthetics. Pick *et al.* showed the volatile anesthetics sevoflurane, isoflurane and halothane all incurred a REM sleep deficit, with halothane also accruing NREM sleep debt [9]. Similarly, Pal *et al.* demonstrated that sevoflurane anesthesia was able to reverse NREM sleep debt, but had no effect on REM sleep [10]. Despite the many similitudes, it is clear that anesthetic-induced unconsciousness is not sleep. There are some fundamental physiological differences present that suggest that the two states are not as close neural correlates as is often described.

## Discussion

### Sleep & anesthesia onset

Sleep is actively generated in the brain and is dependent on homeostatic drive and circadian rhythms. The ease of onset and maintenance of sleep is subject to environmental factors such as temperature, noise, light and chemical stimulants. Once NREM is established, there is regular cycling between this state and REM sleep at approximately 90-min intervals [11]. In contrast, anesthesia is a pharmacologically induced state, the stages of which do not normally show the fluctuation and cycling seen in sleep. Instead there is cycling of brain states in anesthesia, during maintenance at steady-state concentrations, with EEG readings varying among delta, theta, alpha and burst suppression [12,13].

The onset of sleep is known to be regulated by a variety of neurotransmitter systems. In recent years, the prevailing supposition is that sleep–wake regulation is contingent on fast neurotransmitters such as GABA and glutamate [14].

The ventrolateral preoptic area (VLPO) is known to be important in sleep onset. GABAergic neurons project from the VLPO to arousal areas such as the histaminergic tuberomammillary nucleus in the posterior hypothalamus, the serotonergic dorsal raphe and the noradrenergic locus coeruleus [15,16]. Furthermore, certain anesthetics have been shown to stimulate these VLPO neurons to exert their hypnotic effect. Immunohistochemical mouse studies demonstrate an increase in the numbers of active neurons in the VLPO in response to isoflurane and halothane, the same neurons involved in regulating natural sleep [17]. Adenosine is one of the brain's chief somnogens and accumulates as a degradation product of adenosine triphosphate during prolonged intervals of waking [18]. Adenosine binding in the VLPO is associated with increased activity in this region, promoting non-REM sleep [18].

The median preoptic (MnPO) area is another nucleus that contains a large concentration of GABAergic sleep-active neurons and is located dorsal to the third ventricle [18,19]. Like the VLPO region, the MnPO projects neurons that inhibit wake-promoting neurons found in multiple regions of the brain, including the perifornical lateral hypothalamus, dorsal raphe nucleus and the locus coeruleus. Experiments suggest, however, that the MnPO neurons are more active during states of sleep deprivation, compared with the VLPO region, which is more active during sleep [20]. This could highlight differing roles in sleep homeostasis, with the MnPO responding to homeostatic pressure, whereas the VLPO plays a more consolidatory role [18].

It has become increasingly evident that anesthetic agents employ diverse agent-specific mechanisms to achieve their desired effect, some of which correlate with sleep mechanisms.

The sedative dexmedetomidine has a mode of action that is particularly similar to the signatures of natural sleep. Rat studies have shown that administering dexmedetomidine to the locus coeruleus results in an NREM EEG pattern [21], with a similar effect observed in humans [22]. Dexmedetomidine opens K+ channels resulting in hyperpolarization of the noradrenergic neurons of the locus coeruleus, inhibiting their activity [23]. This mimics a similar decrease in activity of locus coeruleus neurons observed during natural sleep [24]. This causes disinhibition of hypothalamic neurons in the preoptic area, which subsequently inhibits midbrain and pontine arousal centres [7]. Recent research has shown that dexmedetomidine mimics NREM stage 3 sleep, which is termed biomimetic sleep. Subjects were also shown to have no impaired performance on subsequent psychomotor tests, compared with zolpidem, a sleep medication that modulates GABA_A_ receptors. This could benefit patients by eliminating cognitive dysfunction postemergence that occurs as a drug side effect [25].

Opioids, such as fentanyl, often used in conjunction with anesthetic agents, decrease arousal by reducing acetylcholine in the medial pontine reticular formation and by binding to opioid receptors at various target sites including the spinal cord and periaqueductal gray [11]. Propofol, a small alkylphenol derivative introduced into practice in the late 1980s, has an anesthetic action that is mediated by GABA_A_ inhibitory action. This hinders release of the arousal-promoting neurotransmitter histamine from the tuberomammillary nucleus in hypothalamus [11].

It has been suggested that the nature of the GABAergic inhibition observed in both sleep and anesthesia has subtle variations. A study by Bjornstrom *et al.* put forth evidence of propofol preferentially targeting the β2 (54) subunit of the GABA_A_ receptor compared to the β2 (56) isomer targeted by endogenous GABA [26]. The subunit distribution varies across the central nervous system, and this may define the site of action of anesthetic agents and natural somnogens. Furthermore, a recent study by Kelz *et al.* has shown the effect of orexin antagonism in anesthetic emergence but a lack of effect on induction [27]. Orexin released from lateral hypothalamic neurons promotes wakefulness. Kelz *et al.* used selective genetic ablation of orexinergic neurons, which causes acquired narcolepsy and resulted in delayed emergence from anesthesia, but without a corresponding effect on anesthetic induction [27]. The results suggest that the process of anesthetic onset and offset are not a reciprocal. Similar conclusions regarding the lack of an inverse relationship between induction and emergence were drawn by Vanini *et al.*, who showed that anesthetic induction in rats is regulated by GABAergic transmission in the pontine reticular formation (PnO) but that emergence is not [28]. Thus, anesthetic and sleep onset may differ with regard to the neurotransmitters and molecular targets in play, as well as the agent in question.

### Sleep & anesthesia maintenance period

#### EEG activity

In both the anesthetic state and natural sleep, thalamocortical signal loops produce EEG activity with sequential transitioning between alpha, theta and delta oscillations, with the exception of agents such as ketamine [29]. At increasing depth of anesthesia and sleep, EEG wave morphologies show changes unique to both sleep and surgical anesthesia. The transition from slow wave sleep to REM sleep corresponds to the switching from delta waves to low voltage, high frequency EEG activity. REM sleep is often described as paradoxical sleep, due to the similarities between its EEG with that of wakefulness. However, there are some differences, a notable one being the decoupling of thalamocortical activity during REM sleep, which is coupled during wakefulness [30]. Partially underlying this process is cholinergic activity from the basal forebrain and pontine tegmental nuclei emanating to the cortex [18]; this complex transition is orchestrated by many systems, however, and is not fully understood.

In contrast, a common EEG pattern characteristic of deep anesthesia is burst suppression, marked by alternating periods of high voltage activity in the brain and periods of no brain activity. Burst suppression is associated with a depletion of the extracellular calcium levels during the burst phase, reducing it to the point where it interferes with calcium dependent neurotransmitter release in synaptic transmission. This is followed by the suppression phase where the calcium levels are restored by neuronal pumps [31]. Theories about what induces burst suppression centre around hyperpolarization of cortical neurons, such as is induced by isoflurane when it acts on GABA_A_ receptors [32]. Other studies have used biophysical computer modeling, which points to decreases in cerebral metabolic rate as being the causative factor for the onset of burst suppression. These studies showed that the decrease in intracellular ATP concentration caused by the inhibitory effect of anesthetics results in increased conductance of K+/ATP channels. This results in an influx of K+ leading to the hyperpolarization that triggers burst suppression [33]. The anesthetic state shows a substantial global reduction in cerebral metabolism when compared to sleep. FDG-PET studies have shown a 54% reduction under general anesthesia compared with at most a 23% reduction during NREM sleep [34]. This supports the notion of a more profound dampening of brain activity compared with sleep. It should also be noted that not all anesthetics produce burst suppression, with dexmedetomidine being an example of such an anesthetic that does not commonly produce this effect [35].

#### Nociception

Complete lack of nociceptive response is a striking feature of the anesthetic state that differs from sleep. Anesthetic agents target ventral and dorsal horn cells to blunt ascending transmission of a noxious stimulus to the thalamus. Opiates used as anesthetic adjuncts further activate descending inhibitory signals from the periaqueductal gray. Sleep can be quickly reversed by stimulating somatosensory receptors or cortex with noxious stimuli, whereas emergence from anesthesia is dependent on drug washout [36]. More recently, however, we have moved beyond the notion that anesthetic emergence is an exclusively pharmacokinetically driven process. Friedman *et al.* used the term *neural inertia* to describe the hysteresis between anesthetic concentrations at induction and emergence [37]. Their animal studies indicated that this hysteresis is not purely due to pharmacokinetics and could be genetically modulated, showing an increased complexity of the conscious state that is more difficult to restore as opposed to disrupting. This theory of neural inertia has been determined to exist in humans as demonstrated by Warnaby *et al.*, through the measurement of saturated slow wave brain activity as an individualized marker for perception loss in anesthesia [38]. They showed a maintenance of slow-wave activity during the process of emergence even with the anesthetic dose at very low hypnotic concentrations, thus indicating the so-called neural inertia on transition to consciousness [38].

#### Reflex suppression & atonia

The immobility component of anesthesia is produced by active inhibition of neural circuits in the brainstem and spinal cord, which leads to suppression of reflex withdrawal from noxious stimuli. For example, both propofol and isoflurane depressed ventral horn neurons, with isoflurane additionally having a significant depressive effect on dorsal horn neurons, although evidence suggests that the ventral neurons are likely to be the critical target [39]. Reflex suppression has been used as a parameter to measure anesthetic potency, or minimum alveolar concentration (MAC), which is defined as the minimum alveolar concentration of inhaled anesthetic at which 50% of people do not move in response to a noxious stimulus [40,41]. Spinal cord reflex loops pose as a protective mechanism and are conserved during natural sleep. Postural control is also diminished in the state of anesthesia which differs from the stereotypical species-specific body postures held during NREM sleep. Furthermore, loss of protective reflexes that are maintained during sleep, such as the cough and gag reflexes, are lost in anesthesia; therefore, airway patency has to be artificially maintained.

#### Autonomic nervous system response

The reflex activation of the sympathetic nervous system in response to noxious stimuli is suppressed by administration of appropriate doses of anesthetic agents measured in MAC-BAR, the minimum concentration required to block the autonomic response [41]. The respiratory response to noxious stimuli consists of raised respiratory rate, tidal volume and increased tendency of breath-holding and laryngospasm. Anesthetic-induced ventilatory and cardiorespiratory depression diminishes these responses that would otherwise reverse natural sleep.

#### Role in cognitive development

Although sleep, like anesthesia, is characterized by amnesia, it is thought to play a key role in memory consolidation and cognitive development. Conversely, anesthetic use exhibits neurotoxicity at the extremes of age, although the etiology is poorly understood. In neonates, the actions of typical general anesthetic agents such as NMDA receptor antagonism and GABA_A_ receptor potentiation have been shown to cause apoptosis and interfere with the neurogenesis process [42]. Postoperative cognitive dysfunction is a common complication of general anesthesia in the elderly with significant associated morbidity and mortality [43]. The underlying mechanisms are thought to be increased vulnerability of the aging brain to insult, causing alterations in calcium homeostasis and acceleration of endogenous neurodegenerative processes [44].

### Sleep & anesthesia emergence

Sleep offset occurs rapidly in a matter of minutes. There is intrinsic resistance to reinitiate sleep immediately after waking but an increased propensity to sleep after deprivation [18]. Conversely, resumption of wakefulness from general anesthesia can take up to hours and, as discussed previously, is not simply a reversal of the induction process. The hypnotic component of anesthesia at a supraspinal level renders a patient unrousable even by vigorous stimuli. During natural sleep, the GABAergic inhibitory postsynaptic potentials, acting in the thalamocortical orientation, are increased, however it is possible for a moderate stimulus to achieve the required cortical depolarization to induce consciousness. Under anesthesia, the dominant effects of enhanced GABA-mediated inhibition and depressed glutamate-mediated excitation on the cortex is such that even high intensities of stimulation are insufficient to increase the firing rate at the neocortex required for the transition into wakefulness [45].

Additionally, re-anesthetizing a patient can be achieved immediately after anesthetic emergence, and this useful technique is carried out in procedures where patients are routinely awakened to test responses intraoperatively, such as during deep brain stimulation electrode implant surgery [46]. Finally, iatrogenic side effects following anesthetic emergence, such as postoperative nausea and vomiting, cardiorespiratory depression and immune function losses stand in stark contrast to the refreshing nature of wakefulness after natural sleep [47–49].

## Conclusion

Sleep research continues to contribute to the pursuit of understanding the process of general anesthesia. This has highlighted both striking similarities and differences. The anesthetic state correlates with physiological endpoints of unconsciousness, analgesia, motor and autonomic reflex suppression and profound depression of brain activity that goes beyond the level observed during natural sleep. These key differences are crucial to successful induction and maintenance of anesthesia and to facilitate surgical intervention. The differences must be accounted for in future comparative studies to further research in the field and may warrant a move away from modeling the anesthetic state on sleep.

## Future perspective

Substantial progress has been made over the past few decades in neuroscience research evaluating the mechanisms of sleep and balanced anesthesia. Anesthetic agents targeting specific molecules are now used to achieve different aspects of the anesthetic state. For example, hypothalamic nuclei have been such targets for anesthetic agents due to their wake-promoting properties. However, our knowledge remains limited, and there remains scope for further research on the relationship between sleep and the anesthetic state. Further research exploring the neuronal mechanisms controlling consciousness in the cortex and thalamus could provide insight into the influence of sleep on anesthesia and vice versa, improving both our clinical understanding and the practice of anesthesia.

**Executive Summary**

- Anesthetic onset and sleep onset differ with regard to the neurotransmitters and molecular targets in play, as well as the anesthetic agent used.
- In recent years, the prevailing supposition is that sleep–wake regulation is contingent on fast neurotransmitters such as GABA and glutamate.
- Thalamocortical signal loops produce electroencephalogram (EEG) activity with sequential transitioning between alpha, theta and delta oscillations in both the anesthetic state and natural sleep, with some exceptions.
- At increasing depth of anesthesia and sleep, EEG wave morphologies show changes unique to both sleep and surgical anesthesia.
- Complete lack of nociceptive response is a striking feature of the anesthetic state that differs from sleep.
- Immobility during anesthesia is produced by active inhibition of neural circuits in the brainstem and spinal cord, which leads to suppression of reflex withdrawal from noxious stimuli and loss of protective reflexes that are maintained during sleep.
- Reflex activation of the sympathetic nervous system in response to noxious stimuli is suppressed during anesthesia, thus diminishing responses that would otherwise reverse natural sleep.
- Although sleep, like anesthesia, is characterized by amnesia, it is thought to play a key role in memory consolidation and cognitive development.
- Sleep offset occurs rapidly in a matter of minutes with natural resistance to reinitiating sleep immediately. Resumption of wakefulness from general anesthesia can take much longer and re-anesthetizing a patient can be achieved immediately after anesthetic emergence.
